# Depression following the initiation of glucagon‐like‐peptide‐1 receptor agonist therapy: A multinational self‐controlled case series study

**DOI:** 10.1111/joim.70098

**Published:** 2026-04-16

**Authors:** Vincent Ka Chun Yan, Zi‐Yang Peng, Vincent Kai Chung Wong, Ian Chi Kei Wong, Esther Wai Yin Chan, Huang‐Tz Ou, Eric Yuk Fai Wan

**Affiliations:** ^1^ Centre for Safe Medication Practice and Research, Department of Pharmacology and Pharmacy, Li Ka Shing Faculty of Medicine The University of Hong Kong Hong Kong SAR China; ^2^ Institute of Clinical Pharmacy and Pharmaceutical Sciences, College of Medicine National Cheng Kung University Tainan Taiwan; ^3^ Department of Pharmacy Queen Mary Hospital Hong Kong SAR China; ^4^ School of Pharmacy, Medical Sciences Division Macau University of Science and Technology Macau China; ^5^ Advanced Data Analytics for Medical Science (ADAMS) Limited Hong Kong SAR China; ^6^ Aston Centre of Excellence in Pharmacotherapy Optimisation and Pharmacoepidemiology Research, Aston Pharmacy School Aston University Birmingham UK; ^7^ Shenzhen Institute of Research and Innovation The University of Hong Kong Shenzhen China; ^8^ Department of Pharmacy, College of Medicine National Cheng Kung University Tainan Taiwan; ^9^ Department of Family Medicine and Primary Care, School of Clinical Medicine, Li Ka Shing Faculty of Medicine The University of Hong Kong Hong Kong SAR China; ^10^ Comprehensive Primary Healthcare Collaboratory, Li Ka Shing Faculty of Medicine The University of Hong Kong Hong Kong SAR China; ^11^ The Institute of Cardiovascular Science and Medicine, Li Ka Shing Faculty of Medicine The University of Hong Kong Hong Kong SAR China

**Keywords:** depression, glucagon‐like‐peptide‐1 receptor agonist, multinational, self‐controlled case series

Dear Editor,

Glucagon‐like‐peptide‐1 receptor agonists (GLP‐1RAs) are increasingly used as adjunctive treatment in type‐2 diabetes and weight management in obesity. However, prior case reports suggested possible neuropsychiatric side effects, potentially linked to glucagon‐like‐peptide‐1 receptor expression in the brain. Consequently, the European Medicines Agency initiated a safety review on the possible association between GLP‐1RAs and suicidal thoughts or self‐harm [1]. The US Food and Drug Administration (FDA) recommends monitoring for depression or suicidal thoughts and includes warnings concerning suicidal behaviour for some GLP1‐RA formulations.

Yet evidence on GLP‐1RAs and depression remains limited and inconsistent. A study using the FDA Adverse Event Reporting System found increased reporting of depression/suicidal events with semaglutide and liraglutide compared with metformin and insulin [2] but could not establish causality and was prone to reporting bias. FDA warnings for liraglutide and semaglutide were based on a pooled post hoc analysis of randomized trials, which showed slightly higher rates of suicidal ideation [3], yet depression rates were similar between liraglutide and placebo (2.1 vs. 2.1 events/100‐person‐years). A cohort study conducted within the Veterans Health Administration in the United States demonstrated no increased risk of depression with GLP‐1RA but had limited generalizability [4]. Meta‐analyses suggested that GLP‐1RAs may protect against depression [5] but were constrained by few available studies and high heterogeneity. Importantly, most studies were conducted in white populations with minimal Asian representation, underscoring the need for further research accounting for ethnic diversity.

We conducted a multinational study using data from Hong Kong, the United Kingdom and Taiwan. Supporting Information provides details of the databases and analyses. Using a self‐controlled case series (SCCS) design, we assessed the risk of incident depression (Table S1) after GLP‐1RA initiation. SCCS compares periods within the same individual, eliminating time‐invariant confounders such as genetics, socioeconomic status and baseline disease severity [6]. Only exposed cases are required; therefore, we included individuals with a first depression diagnosis and received a first‐ever GLP‐1RA prescription during the study period (Hong Kong: 2008–2023, the United Kingdom: 2008–2021, Taiwan: 2012–2020), excluding individuals aged <18 or with prior depression. Follow‐up began 1 year before GLP‐1RA initiation (or study start, or 1 year after registration in database), until the earliest of 2 years after GLP‐1RA initiation, death or study end (or transfer out from database). Observation time was divided into risk periods: Days 0–179, 180–364 and 365–729 after initiation, and the baseline period: Day‐365 to Day‐1 before initiation, with a 180‐day pre‐exposure window (Day‐180 to Day‐1) before initiation to account for event‐dependent exposure (Fig. S2). Conditional Poisson regression was used to estimate incidence rate ratios, adjusting for age as a time‐varying confounder. Pooled incidence rate ratios across study sites were calculated using random‐effects meta‐analysis, with heterogeneity assessed using *I* ^2^.

A total of 2212 depression cases across three study sites were included in the analyses (Fig. S1). The mean (SD) ages at the beginning of the observation period were between 40.72 (14.67) and 53.85 (14.58), and 36.2%–53.8% were male (Table S1). Pooling all study sites, there was no significant difference in risk of incident depression after GLP‐1RA initiation compared to the non‐exposure period (Table 1). Findings were consistent across all study sites, including analyses stratified by sex and individual GLP‐1RAs, and in all sensitivity analyses (Tables S2–S4).

**Table 1:** Risk of incident depression after initiation of glucagon‐like‐peptide‐1 receptor agonist (GLP‐1RA) therapy.

	No. of events	Follow‐up (person‐years)	Crude incidence (per person‐year)^b^	Incident rate ratio (95% CI)
**Pooled (all sites)** ^a^				
Pre‐exposure period	414	1068.6	0.387	0.943 (0.819–1.087)
0–179 days after initiation	409	1051.1	0.389	0.987 (0.795–1.226)
180–364 days after initiation	350	1037.4	0.337	0.904 (0.717–1.140)
365–729 days after initiation	604	1817.7	0.332	0.968 (0.721–1.299)
Non‐exposure period	435	1036.2	0.420	(Ref)
**Hong Kong**				
Pre‐exposure period	10	39.4	0.254	0.655 (0.295–1.450)
0–179 days after initiation	15	39.4	0.381	1.010 (0.489–2.086)
180–364 days after initiation	12	39.2	0.306	0.848 (0.391–1.840)
365–729 days after initiation	27	68.0	0.397	1.176 (0.609–2.272)
Non‐exposure period	16	40.2	0.398	(Ref)
**The United Kingdom**				
Pre‐exposure period	147	385.2	0.382	0.891 (0.710–1.117)
0–179 days after initiation	144	387.6	0.372	0.866 (0.687–1.090)
180–364 days after initiation	127	382.1	0.332	0.803 (0.631–1.022)
365–729 days after initiation	226	691.2	0.327	0.805 (0.645–1.005)
Non‐exposure period	155	371.0	0.418	(Ref)
**Taiwan**				
Pre‐exposure period	257	643.9	0.399	1.000 (0.830–1.205)
0–179 days after initiation	250	624.1	0.401	1.120 (0.891–1.408)
180–364 days after initiation	211	616.1	0.343	1.061 (0.795–1.417)
365–729 days after initiation	351	1058.5	0.332	1.168 (0.806–1.694)
Non‐exposure period	264	625.0	0.422	(Ref)

^a^ *I* ^2^ for pooled incident rate ratio = 35% (Days 0–179), 28% (Days 180–364), 46% (Days 365–729). Additionally, there were 414 events during the pre‐exposure period (Hong Kong: 10, the United Kingdom: 147, Taiwan: 257) as displayed in this summary table.

^b^ The reported crude incidence rates should only be interpreted as relative to incidence rates in other risk periods or the non‐exposure period and do not represent the absolute incidence rate in the general population, as this is a case‐only study without information on non‐users or people without the outcome.

Our study supports the neutral effect of GLP1‐RA therapy on incident depression, which is consistent with a few large‐scale studies. A target trial emulation using US National Medicare data found a slightly lower depression risk with GLP‐1RA versus DPP‐4 inhibitor (HR 0.90 [0.82–0.98]) [7]. Another UK study comparing GLP‐1RA versus sulfonylurea observed no increased risk of depression or self‐harm (HR 1.25 [0.63–2.50]) [8]. A meta‐analysis of randomized trials (*N* = 84,713) reported no increased risk of depression (MH‐OR 0.96 [0.68–1.35]) [9]. Our findings may be explained by the divergent anxiogenic and antidepressant effects of GLP‐1RAs [10], with some hypotheses of potential neurological benefits based on biological mechanisms.

Our study addressed limitations of prior research by applying the SCCS design to eliminate unmeasured time‐invariant confounding, which is challenging to account for in traditional cohort designs, and including real‐world populations from three countries with diverse ethnicities and clinical practices. A few limitations of this study include residual time‐varying confounding (e.g., due to COVID‐19 or co‐medications, despite adjustment for age), limited sample size for some subgroups, heterogeneity due to differences in clinical practice and healthcare access across sites, possible underdiagnosis of depression and inability to consider recurrent depression events.

To conclude, this multinational SCCS study found no significant difference in depression risk following GLP‐1RA initiation. Routine monitoring of patients’ mental health is still suggested to ensure the rational and safe use of GLP1‐RAs.

## Author contributions

Eric Yuk Fai Wan and Ian Chi Kei Wong conceived the study. Vincent Ka Chun Yan designed the study, analysed and interpreted the data and wrote the manuscript. Zi‐Yang Peng designed the study, analysed and interpreted the data and edited the manuscript. Huang‐Tz Ou provided study materials, designed the study, interpreted the data and wrote the manuscript. Eric Yuk Fai Wan provided study materials, designed the study, interpreted the data and reviewed the manuscript. Ian Chi Kei Wong and Esther Wai Yin Chan provided the study materials. Vincent Kai Chung Wong provided administrative support. All authors approved the final manuscript. Huang‐Tz Ou and Eric Yuk Fai Wan are guarantors.

## Conflict of interest

Eric Yuk Fai Wan has received research grants from the Health Bureau, the Hong Kong Research Grants Council, Narcotics Division, Security Bureau, Social Welfare Department, Labour and Welfare Bureau of the Government of the Hong Kong SAR and National Natural Science Foundation of China; serves on member of Core Team for Expert Group on Drug Registration of Pharmacy and Poisons Board and is the director of Advance Data Analytics for Medical Science (ADAMS) Limited (HK). These are outside the submitted work. Ian Chi Kei Wong received research grants outside of submitted work from European Commission, Research Grants Council of the Hong Kong Special Administrative Region of the People's Republic of China, Health and Medical Research Fund of the Government of the Hong Kong Special Administrative Region of the People's Republic of China and Amgen; consulting fees from IQVIA and World Health Organization; served as a member of Pharmacy and Poisons Board of Hong Kong and was the founder and director of Therakind Limited (UK). He is the non‐executive director of Jacobson Pharma Corp. Ltd. in Hong Kong and director of Advance Data Analytics for Medical Science (ADAMS) Limited (HK), Healthcare Innovation Technology Service (HITS) Limited (UK) and OCUS Innovation Limited (HK, Ireland and UK). He received honorarium from Takeda as a speaker of continuous professional education study day. EWYC reports grants from the Hong Kong Research Grants Council of the Government of the Hong Kong SAR, Research Fund Secretariat of the Food and Health Bureau, National Natural Science Fund of China, Bayer, Bristol‐Myers Squibb, Pfizer, Janssen, Amgen, Takeda, RGA Reinsurance Company, AstraZeneca, Narcotics Division of the Security Bureau of the Hong Kong Special Administrative Region, Innovation and Technology Commission of the Government of the Hong Kong Special Administrative Region, Novartis, and honorarium from Hospital Authority, outside the submitted work.

## Funding information

The National Science and Technology Council of Taiwan (grant number NSTC 112‐2628‐B‐006‐008‐MY3) (recipient: Huang‐Tz Ou) and National Natural Science Foundation of China (NSFC) Excellent Young Scientists Fund (Hong Kong and Macau) (Ref No. 82222902) (recipient: Eric Yuk Fai Wan).

## Disclosure

The funders had no role in the design and conduct of the study; collection, management, analysis and interpretation of the data; preparation, review or approval of the manuscript; and decision to submit the manuscript for publication.

## Ethics statement

This study was approved by the Institutional Review Board from the three study sites (HK: UW 24‐122, Taiwan: A‐EX‐109‐035, UK: 24SRC001). The informed consent was waived because this study did not involve individual patients.

## Consent

Not applicable.

## Supporting information




**Table S1**: Characteristics of cases included in the SCCS.
**Table S2**: Subgroup analyses pooled results across all sites.
**Table S3**: Sensitivity analyses pooled results across all sites.
**Table S4**: Subgroup and sensitivity analyses results of each individual site.
**Figure S1**: Flowchart for selection of SCCS cases.
**Figure S2**: Study design.

## Data Availability

Data may be obtained from a third party and are not publicly available. Taiwan site: Raw data were generated at Taiwan's National Health Insurance Research Database. Derived data supporting the findings of this study are available from the corresponding author (Huang‐Tz Ou) on request. HK and UK sites: The underlying data supporting this analysis cannot be directly shared as they are obtained under licence from IQVIA (for UK data) and owned by Hospital Authority of Hong Kong (for HK data), and data custodians have not given permission for sharing.
