# Supplementary material for: Depression following the initiation of glucagon‐like‐peptide‐1 receptor agonist therapy: A multinational self‐controlled case series study
Source: J Intern Med. 2026 Apr 16;299(6):776–80. doi: 10.1111/joim.70098 (PMC13137393; doi:10.1111/joim.70098)
Supplement: Supplementary file 1 — Table S1: Characteristics of cases included in the SCCS. Table S2: Subgroup analyses pooled results across all sites. Table S3: Sensitivity analyses pooled results across all sites. Table S4: Subgroup and sensitivity analyses results of each individual site. Figure S1: Flowchart for selection of SCCS cases. Figure S2: Study design. [file JOIM-299-776-s001.docx]

**Supplementary Methods**

*Data sources*

The Clinical Data Analysis and Reporting System (CDARS) is a territory-wide electronic medical records database from Clinical Management System (CMS) of the Hospital Authority (HA) of HK. HA is the statutory administrative organization managing the public healthcare sector in HK. HA is funded by the HKSAR Government and provides subsidised clinical services to over 7.4 million HK residents, covering approximately 80% of all hospital admissions in HK.^1^ The CMS provides real-time information to support day-to-day patient care operations across all public hospitals and clinics. CDARS contains data covering all aspects of patient care, including demographics, diagnoses, procedures, prescriptions, laboratory tests, inpatient admissions, and outpatient and emergency department attendances. CDARS is an internationally recognised database with territory-wide population coverage and comprehensive coverage of various aspects of patient care, and has been used in numerous healthcare big data research studies.^2-7^ Previous studies demonstrated a high degree of coding accuracy for a variety of outcomes.^3,6,8^

The IQVIA Medical Research Data (IMRD)-UK database, which incorporates data from The Health Improvement Network (THIN), a Cegedim Database,^9^ is a nationwide database of primary care records in the UK that includes around 6% of the total UK population. This work used de-identified data provided by patients as part of their routine primary care from within this database. Previous studies have demonstrated the validity of the database for pharmacoepidemiologic studies and generalisability to the UK population.^10,11^ The IMRD-UK database includes data recorded in primary care settings on demographic information, lifestyle information, medical diagnoses and procedures (recorded in read codes), laboratory test values, and prescribing information.

The National Health Insurance Research Database (NHIRD) is a claims database in Taiwan. It contains anonymized individual-level claims-based data of healthcare services (e.g., outpatient and emergency room visits, hospitalizations, and medication prescriptions) of beneficiaries enrolled in the National Health Insurance program, which includes over 99% of Taiwan’s population.^12^

*Study design*

The SCCS study design is a within-individual comparison based on a case-only approach^13^. In an SCCS design, participants serve as their own controls, and relative risk is estimated based on within-person rather than between-person comparisons, by comparing the rate of outcome events between different risk windows and the baseline period of the same individual. The major advantage of SCCS over other between-individual comparison study designs (such as a cohort study) is that it eliminates both measured and unmeasured time-invariant confounders that vary between individuals, such as family history, genetic factors, socioeconomic status, and underlying disease severity^13^. Furthermore, time-varying factors, such as age and season, could be adjusted for. The SCCS study design has been applied in numerous post-marketing pharmacovigilance studies evaluating rare adverse effects of medications and vaccines.^8,14-18^

There are a few major assumptions of the SCCS design that need to be met in this study. Firstly, events should arise independently within individuals or, if non-recurrent, be uncommon. Becausewe could not rule out the possibility that a first depression episode increases the chance of a second, nor reliably differentiate a new depression episode versus follow-up care of an existing depression episode, only the first-ever depression event was considered in this study, and cases with a history of depression before start of observation period were excluded to meet this assumption. Secondly, events should not influence the probability of subsequent exposure. During the study period, depression had not been considered as a contraindication for prescribing or discontinuing GLP-1RAs according to international guidelines. Therefore, the presence of pre-existing depression should not influence the likelihood of initiating GLP-1RA therapy. Nevertheless, a pre-exposure period of 180 days was added in the main analyses to account for the possibility of event-dependent exposure. Thirdly, events shall not censor the observation period. The fatality rate of depression is relatively low. Sensitivity analysis was also conducted by excluding patients who died during the observation period to mitigate this possibility.

*Study population*

Because this is a self-controlled case series analysis, only exposed cases (i.e., patients with GLP-1RA exposure and incident diagnosis of depression within two years after GLP-1RA initiation during the study period) need to be analysed. Therefore, individuals with their first diagnosis of depression and who initiated any GLP-1RA (first-ever prescription) during the study period (HK: 2008-2023, UK: 2008-2021, Taiwan: 2012-2020) were included, and individuals aged less than 18 years or who had a history of depression before the start of the study period were excluded. Additionally, for UK data, only individuals with at least one year of up-to-standard data record in the database were included.

*Exposure and outcomes*

The exposure of interest in this study was GLP-1RA use, defined as any prescriptions of GLP-1RAs. The primary outcome was the incident diagnosis of depression, defined as the first diagnosis record of depression identified using the following diagnosis codes in each database.

| **Study site** | **Operational definition of depression** |
| --- | --- |
| Hong Kong | ICD-9 code: 296.2, 296.3, 300.4, 311 |
| United Kingdom | Read codes: Eu20400, Eu33214, E113700, Eu32312, Eu33y00, E118.00, 9H91.00, Eu32.13, E112.11, E002100, Eu33.14, E113300, Eu32000, E113z00, Eu33z11, Eu33200, E112.14, E112200, E113100, E112400, Eu33211, E004300, E112000, Eu32800, Eu32311, Eu32212, Eu33316, 9k4..00, E113.11, Eu32400, Eu32z00, Eu33311, Eu33315, Eu41200, E113600, 9Ov..00, 9H90.00, E11z200, Eu33300, Eu32z13, Eu34112, Eu32500, E113.00, E113000, Eu32314, Eu32700, E112.00, Eu33z00, Eu33312, Eu25100, 9HA0.00, Eu32600, E112300, 9Ov2.00, Eu32.00, E112.13, Eu32313, Eu33212, Eu32.12, 8CAa.00, E001300, Eu32300, 9kQ..00, E11..12, Eu32z11, E113500, Eu32.11, Eu33400, Eu25112, E130.00, Eu32y12, 1465.00, E113400, E112500, 9Ov1.00, Eu33.13, 9H92.00, Eu33314, E112z00, E112100, E2B..00, E291.00, 9Ov0.00, Eu33.11, Eu34114, Eu33100, E200300, Eu34100, Eu32211, Eu32213, E130.11, 212S.00, E113200, E112.12, Eu33313, Eu33.12, Eu33213, Eu34113, Eu33.15, 8HHq.00, Eu33.00, E135.00, Eu34111, Eu32y00, E112600, Eu32100, 9Ov4.00, 8BK0.00, Eu41211, Eu32A00, Eu32y11, Eu32z12, 9k40.00, Eu32900, Eu33000, 9Ov3.00, Eu32z14, Eu25111, Eu32200, E11y200, E2B1.00 |
| Taiwan | ICD-9-CM code: 296.2, 296.3, 300.4, 311  ICD-10-CM code: F32, F33, F34.1 |

*Definitions of observation period and risk periods*

Individuals were followed up from one year before GLP-1RA initiation (or the start of study period, or one year after patient’s registration to general practice [for UK data only], whichever is later), until the earliest of i) two years after GLP-1RA initiation, ii) the date of death, iii) the end of study period (or date of transfer out from general practice for UK data). RCTs have reported a low rate of depression in liraglutide treatment groups over periods of up to 56 weeks.^19^ Therefore, a two-year observation window following the initiation of GLP-1RA therapy was considered sufficient to capture any potential risk of depression associated with the treatment. Person-time during the observation period was divided into discrete risk windows based on the number of days after GLP-1RA initiation, as follows: Days 0-179, Days 180-364, or Days 365-729 after the start date of the first-ever prescription of GLP-1RA. A pre-exposure period of 180 days before GLP-1RA initiation was added to account for the possibility of event-dependent exposure, with sensitivity analyses varying the duration of the pre-exposure period. Periods of follow-up that are not risk periods were classified as non-exposure (reference) periods (**Figure 2**).

*Statistical analysis*

Time-varying confounders, including age (five-year band between 30 and 80 years), were adjusted in the SCCS model. Incidence rates for outcomes in each risk window were reported. Conditional Poisson regression was used to compare the incidence rates in each risk window with reference periods. Incidence rate ratios (IRRs) and 95% confidence intervals (CIs) for each risk window were estimated. Pooled IRR with 95% CI was calculated separately for each risk period based on a meta-analysis across all study sites with a random effects model, and the heterogeneity of study estimates was assessed using I^2^. Analyses stratified by sex and individual GLP-1RA drugs were conducted. Sensitivity analyses were conducted by: i) ending the observation period at 30 days after discontinuation of GLP-1RA (if earlier); ii) excluding patients who died during the observation period; iii) treating day 0 as a separate risk period; iv) excluding events that happen on the same day of GLP-1RA initiation; v) varying the duration of pre-exposure risk period as 90 days. All analyses were conducted in R using the “SCCS” and “metafor” packages and SAS [for Taiwan site only]. A *p*-value of less than 0.05 was considered statistically significant.

*Ethics approval*

This study was approved by the Institutional Review Board of the University of Hong Kong/Hospital Authority Hong Kong West Cluster (UW 24-122), the IMRD Scientific Review Committee (24SRC001), and the Research Ethics Committee (REC) in Taiwan (A-EX-109-035).

*References*

1. HKSAR Government Health Bureau. Hong Kong Healthcare Systems and Healthcare Professionals. Accessed 21 May 2020, <https://www.healthbureau.gov.hk/download/press_and_publications/otherinfo/180500_sr/e_ch1.pdf>

2. Lai EC-C, Man KKC, Chaiyakunapruk N, et al. Brief Report: Databases in the Asia-Pacific Region: The Potential for a Distributed Network Approach. *Epidemiology*. 2015;26(6)

3. Law SWY, Lau WCY, Wong ICK, et al. Sex-Based Differences in Outcomes of Oral Anticoagulation in Patients With Atrial Fibrillation. *J Am Coll Cardiol*. Jul 17 2018;72(3):271-282. doi:10.1016/j.jacc.2018.04.066

4. Lau WCY, Cheung C-L, Man KKC, et al. Association Between Treatment With Apixaban, Dabigatran, Rivaroxaban, or Warfarin and Risk for Osteoporotic Fractures Among Patients With Atrial Fibrillation. *Annals of Internal Medicine*. 2020/07/07 2020;173(1):1-9. doi:10.7326/M19-3671

5. Man KKC, Chan EW, Ip P, et al. Prenatal antidepressant use and risk of attention-deficit/hyperactivity disorder in offspring: population based cohort study. *BMJ*. 2017;357:j2350. doi:10.1136/bmj.j2350

6. Man KKC, Coghill D, Chan EW, et al. Association of Risk of Suicide Attempts With Methylphenidate Treatment. *JAMA Psychiatry*. 2017;74(10):1048-1055. doi:10.1001/jamapsychiatry.2017.2183

7. Chai Y, Luo H, Wong GHY, et al. Risk of self-harm after the diagnosis of psychiatric disorders in Hong Kong, 2000&#x2013;10: a nested case-control study. *The Lancet Psychiatry*. 2020;7(2):135-147. doi:10.1016/S2215-0366(20)30004-3

8. Wong AYS, Wong ICK, Chui CSL, et al. Association Between Acute Neuropsychiatric Events and Helicobacter pylori Therapy Containing Clarithromycin. *JAMA Internal Medicine*. 2016;176(6):828-834. doi:10.1001/jamainternmed.2016.1586

9. The Health Improvement Network. 12 Dec, 2023. <https://www.the-health-improvement-network.com/>

10. Lewis JD, Schinnar R, Bilker WB, Wang X, Strom BL. Validation studies of the health improvement network (THIN) database for pharmacoepidemiology research. *Pharmacoepidemiol Drug Saf*. Apr 2007;16(4):393-401. doi:10.1002/pds.1335

11. Blak BT, Thompson M, Dattani H, Bourke A. Generalisability of The Health Improvement Network (THIN) database: demographics, chronic disease prevalence and mortality rates. *Inform Prim Care*. 2011;19(4):251-5. doi:10.14236/jhi.v19i4.820

12. Hsieh CY, Su CC, Shao SC, et al. Taiwan's National Health Insurance Research Database: past and future. *Clin Epidemiol*. 2019;11:349-358. doi:10.2147/clep.S196293

13. Farrington P, Whitaker H, Weldeselassie YG. *Self-controlled case series studies: a modelling guide with R*. CRC Press; 2018.

14. Wan EYF, Chui CSL, Lai FTT, et al. Bell's palsy following vaccination with mRNA (BNT162b2) and inactivated (CoronaVac) SARS-CoV-2 vaccines: a case series and nested case-control study. *The Lancet Infectious Diseases*. 2022;22(1):64-72. doi:10.1016/S1473-3099(21)00451-5

15. Wan EYF, Chui CSL, Wang Y, et al. Herpes zoster related hospitalization after inactivated (CoronaVac) and mRNA (BNT162b2) SARS-CoV-2 vaccination: A self-controlled case series and nested case-control study. *The Lancet Regional Health – Western Pacific*. 2022;21doi:10.1016/j.lanwpc.2022.100393

16. Man KKC, Lau WCY, Coghill D, et al. Association between methylphenidate treatment and risk of seizure: a population-based, self-controlled case-series study. *Lancet Child Adolesc Health*. Jun 2020;4(6):435-443. doi:10.1016/S2352-4642(20)30100-0

17. Chui CSL, Fan M, Wan EYF, et al. Thromboembolic events and hemorrhagic stroke after mRNA (BNT162b2) and inactivated (CoronaVac) covid-19 vaccination: A self-controlled case series study. *eClinicalMedicine*. 2022;50doi:10.1016/j.eclinm.2022.101504

18. Wan EYF, Wang Y, Chui CSL, et al. Safety of an inactivated, whole-virion COVID-19 vaccine (CoronaVac) in people aged 60 years or older in Hong Kong: a modified self-controlled case series. *The Lancet Healthy Longevity*. 2022;3(7):e491-e500.

19. O'Neil PM, Aroda VR, Astrup A, et al. Neuropsychiatric safety with liraglutide 3.0 mg for weight management: Results from randomized controlled phase 2 and 3a trials. *Diabetes Obes Metab*. Nov 2017;19(11):1529-1536. doi:10.1111/dom.12963

**Supplementary Table 1. Characteristics of cases included in the SCCS**

|  | **Hong Kong** | **United Kingdom** | **Taiwan** |
| --- | --- | --- | --- |
| Number of cases | 80 | 799 | 1333 |
| Age, years – mean (SD) | 40.72 (14.67) | 48.66 (10.94) | 53.85 (14.58) |
| Sex, male – no. (%) | 29 (36.2) | 430 (53.8) | 507 (38.0) |
| Duration of follow-up, years – mean (SD) | 2.82 (0.38) | 2.77 (0.47) | 2.70 (0.47) |
| Duration of GLP-1RA treatment, years – mean (SD) | 1.59 (1.31) | 1.68 (1.82) | 0.94 (1.03) |
| Died during observation period – no. (%) | 2 (2.5) | 5 (0.6) | 88 (6.6) |
| GLP-1RA – no. (%) | |  |  |
| dulaglutide | 25 (31.2) | 74 (9.3) | 577 (43.2) |
| exenatide | 20 (25.0) | 333 (41.7) | 0 (0.0) |
| liraglutide | 23 (28.7) | 325 (40.7) | 750 (56.2) |
| lixisenatide | 3 (3.8) | 28 (3.5) | 0 (0.0) |
| semaglutide | 9 (11.2) | 39 (4.9) | 6 (0.4) |

**Supplementary Table 2. Subgroup analyses – pooled results across all sites**

|  | **No. of events** | **Follow-up (person-years)** | **Crude incidence (per person-year)** | **Incident rate ratio (95% CI)** |
| --- | --- | --- | --- | --- |
| **Sex** |  |  |  |  |
| *Male* |  |  |  |  |
| Pre-exposure period | 176 | 465.7 | 0.378 | 0.948 (0.763-1.177) |
| 0-179 days after initiation | 179 | 458.5 | 0.390 | 0.846 (0.416-1.719) |
| 180-364 days after initiation | 167 | 452.4 | 0.369 | 0.992 (0.749-1.314) |
| 365-729 days after initiation | 258 | 790.0 | 0.327 | 0.890 (0.599-1.322) |
| Non-exposure period | 186 | 454.3 | 0.409 | (Ref) |
| *Female* |  |  |  |  |
| Pre-exposure period | 238 | 602.9 | 0.395 | 0.940 (0.777-1.136) |
| 0-179 days after initiation | 230 | 592.6 | 0.388 | 0.971 (0.782-1.205) |
| 180-364 days after initiation | 183 | 585.0 | 0.313 | 0.814 (0.588-1.127) |
| 365-729 days after initiation | 346 | 1027.6 | 0.337 | 0.964 (0.702-1.324) |
| Non-exposure period | 249 | 581.9 | 0.428 | (Ref) |
| **Drug** |  |  |  |  |
| *Dulaglutide* |  |  |  |  |
| Pre-exposure period | 135 | 330.7 | 0.408 | 0.944 (0.731-1.219) |
| 0-179 days after initiation | 125 | 314.7 | 0.397 | 0.900 (0.613-1.322) |
| 180-364 days after initiation | 113 | 301.3 | 0.375 | 0.941 (0.653-1.355) |
| 365-729 days after initiation | 152 | 475.4 | 0.320 | 0.796 (0.522-1.212) |
| Non-exposure period | 151 | 331.7 | 0.455 | (Ref) |
| *Exenatide* |  |  |  |  |
| Pre-exposure period | 55 | 169.0 | 0.325 | 0.805 (0.557-1.164) |
| 0-179 days after initiation | 68 | 172.5 | 0.394 | 0.976 (0.685-1.390) |
| 180-364 days after initiation | 59 | 174.1 | 0.339 | 0.847 (0.585-1.228) |
| 365-729 days after initiation | 111 | 330.5 | 0.336 | 0.855 (0.537-1.361) |
| Non-exposure period | 60 | 155.2 | 0.386 | (Ref) |
| *Liraglutide* |  |  |  |  |
| Pre-exposure period | 212 | 528.7 | 0.401 | 1.117 (0.908-1.373) |
| 0-179 days after initiation | 199 | 527.0 | 0.378 | 1.105 (0.871-1.403) |
| 180-364 days after initiation | 167 | 531.5 | 0.314 | 0.978 (0.743-1.287) |
| 365-729 days after initiation | 329 | 967.1 | 0.340 | 1.159 (0.873-1.540) |
| Non-exposure period | 191 | 509.3 | 0.375 | (Ref) |
| *Lixisenatide* |  |  |  |  |
| Pre-exposure period | 3 | 13.5 | 0.223 | 0.282 (0.077-1.031) |
| 0-179 days after initiation | 2 | 13.2 | 0.152 | 0.189 (0.041-0.877) |
| 180-364 days after initiation | 7 | 12.6 | 0.555 | 0.725 (0.260-2.024) |
| 365-729 days after initiation | 6 | 22.9 | 0.262 | 0.368 (0.121-1.121) |
| Non-exposure period | 10 | 14.8 | 0.678 | (Ref) |
| *Semaglutide* |  |  |  |  |
| Pre-exposure period | 7 | 20.7 | 0.337 | 0.351 (0.143-0.863) |
| 0-179 days after initiation | 14 | 22.3 | 0.629 | 0.650 (0.321-1.320) |
| 180-364 days after initiation | 4 | 16.4 | 0.244 | 0.278 (0.091-0.846) |
| 365-729 days after initiation | 6 | 18.8 | 0.320 | 0.413 (0.148-1.152) |
| Non-exposure period | 23 | 25.2 | 0.912 | (Ref) |

**Supplementary Table 3. Sensitivity analyses – pooled results across all sites**

|  | **No. of events** | **Follow-up (person-years)** | **Crude incidence (per person-year)** | | **Incident rate ratio (95% CI)** |
| --- | --- | --- | --- | --- | --- |
| *Censor on GLP-1RA discontinuation* | |  |  | |  |
| Pre-exposure period | 414 | 973.1 | 0.425 | | 0.945 (0.819-1.089) |
| 0-179 days after initiation | 394 | 947.3 | 0.416 | | 0.969 (0.811-1.159) |
| 180-364 days after initiation | 301 | 879.2 | 0.342 | | 0.869 (0.700-1.078) |
| 365-729 days after initiation | 470 | 1413.8 | 0.332 | | 0.968 (0.648-1.448) |
| Non-exposure period | 435 | 944.8 | 0.460 | | (Ref) |
| *Exclude death cases* |  |  |  | |  |
| Pre-exposure period | 390 | 1022.9 | 0.381 | | 0.938 (0.811-1.086) |
| 0-179 days after initiation | 396 | 1020.4 | 0.388 | | 0.980 (0.800-1.200) |
| 180-364 days after initiation | 334 | 1007.2 | 0.332 | | 0.871 (0.700-1.084) |
| 365-729 days after initiation | 585 | 1754.0 | 0.334 | | 0.951 (0.717-1.262) |
| Non-exposure period | 412 | 992.4 | 0.415 | | (Ref) |
| *Exclude depression events on the same day of GLP-1RA initiation* | | | | | |
| Pre-exposure period | 157 | 419.2 | 0.375 | | 0.871 (0.700-1.083) |
| 0-179 days after initiation | 147 | 421.4 | 0.349 | | 0.812 (0.649-1.017) |
| 180-364 days after initiation | 139 | 415.7 | 0.334 | | 0.805 (0.639-1.014) |
| 365-729 days after initiation | 253 | 750.1 | 0.337 | | 0.855 (0.640-1.142) |
| Non-exposure period | 171 | 405.6 | 0.422 | | (Ref) |
| *No pre-exposure period* | | | |  |  |
| 0-179 days after initiation | 409 | 1051.1 | 0.389 | | 1.024 (0.870-1.205) |
| 180-364 days after initiation | 350 | 1037.4 | 0.337 | | 0.932 (0.790-1.099) |
| 365-729 days after initiation | 604 | 1817.7 | 0.332 | | 1.023 (0.781-1.340) |
| Non-exposure period | 849 | 2104.8 | 0.403 | | (Ref) |
| *Pre-exposure period of 90 days* |  |  |  | |  |
| Pre-exposure period | 201 | 537.6 | 0.374 | | 0.931 (0.789-1.098) |
| 0-179 days after initiation | 409 | 1064.8 | 0.384 | | 0.973 (0.832-1.139) |
| 180-364 days after initiation | 350 | 1047.9 | 0.334 | | 0.886 (0.751-1.046) |
| 365-729 days after initiation | 604 | 1817.7 | 0.332 | | 0.952 (0.745-1.215) |
| Non-exposure period | 648 | 1567.1 | 0.414 | | (Ref) |
| *Adjust for season* |  |  |  | |  |
| Pre-exposure period | 414 | 1068.6 | 0.387 | | 0.919 (0.794-1.063) |
| 0-179 days after initiation | 409 | 1051.1 | 0.389 | | 0.919 (0.777-1.086) |
| 180-364 days after initiation | 350 | 1037.4 | 0.337 | | 0.843 (0.697-1.020) |
| 365-729 days after initiation | 604 | 1817.7 | 0.332 | | 0.861 (0.712-1.041) |
| Non-exposure period | 435 | 1036.2 | 0.420 | | (Ref) |

**Supplementary Table 4. Subgroup and sensitivity analyses –results of each individual site**

**Hong Kong**

|  | **No. of events** | **Follow-up (person-years)** | **Crude incidence (per person-year)** | | **Incident rate ratio (95% CI)** |
| --- | --- | --- | --- | --- | --- |
| **Sex** |  |  |  | |  |
| *Male* |  |  |  | |  |
| Pre-exposure period | 4 | 14.3 | 0.280 | | 0.415 (0.130-1.328) |
| 0-179 days after initiation | 2 | 14.3 | 0.140 | | 0.213 (0.046-0.980) |
| 180-364 days after initiation | 4 | 14.6 | 0.275 | | 0.425 (0.131-1.373) |
| 365-729 days after initiation | 9 | 24.1 | 0.373 | | 0.608 (0.237-1.562) |
| Non-exposure period | 10 | 14.7 | 0.681 | | (Ref) |
| *Female* |  |  |  | |  |
| Pre-exposure period | 6 | 25.1 | 0.239 | | 1.057 (0.336-3.324) |
| 0-179 days after initiation | 13 | 25.1 | 0.517 | | 2.477 (0.895-6.856) |
| 180-364 days after initiation | 8 | 24.6 | 0.325 | | 1.678 (0.549-5.128) |
| 365-729 days after initiation | 18 | 43.9 | 0.410 | | 2.233 (0.822-6.066) |
| Non-exposure period | 6 | 25.5 | 0.235 | | (Ref) |
| **Drug** |  |  |  | |  |
| *Dulaglutide* |  |  |  | |  |
| Pre-exposure period | 2 | 12.3 | 0.162 | | 0.279 (0.058-1.349) |
| 0-179 days after initiation | 4 | 12.3 | 0.325 | | 0.551 (0.160-1.894) |
| 180-364 days after initiation | 5 | 12.7 | 0.395 | | 0.670 (0.211-2.126) |
| 365-729 days after initiation | 7 | 21.0 | 0.334 | | 0.611 (0.206-1.816) |
| Non-exposure period | 7 | 12.7 | 0.553 | | (Ref) |
| *Exenatide* |  |  |  | |  |
| Pre-exposure period | 2 | 9.9 | 0.203 | | 0.663 (0.111-3.972) |
| 0-179 days after initiation | 4 | 9.9 | 0.406 | | 1.584 (0.339-7.394) |
| 180-364 days after initiation | 2 | 10.1 | 0.198 | | 0.768 (0.122-4.833) |
| 365-729 days after initiation | 9 | 19.0 | 0.473 | | 1.707 (0.425-6.857) |
| Non-exposure period | 3 | 9.8 | 0.306 | | (Ref) |
| *Liraglutide* |  |  |  | |  |
| Pre-exposure period | 4 | 11.3 | 0.353 | | 2.056 (0.377-11.223) |
| 0-179 days after initiation | 4 | 11.3 | 0.353 | | 1.972 (0.360-10.791) |
| 180-364 days after initiation | 4 | 10.7 | 0.375 | | 2.493 (0.441-14.087) |
| 365-729 days after initiation | 9 | 20.2 | 0.446 | | 3.141 (0.644-15.305) |
| Non-exposure period | 2 | 11.6 | 0.172 | | (Ref) |
| *Lixisenatide* |  |  |  | |  |
| Pre-exposure period | 2 | 1.5 | 1.353 | | - |
| 0-179 days after initiation | 1 | 1.5 | 0.676 | | - |
| 180-364 days after initiation | 0 | 1.5 | 0.000 | | - |
| 365-729 days after initiation | 0 | 3.0 | 0.000 | | - |
| Non-exposure period | 0 | 1.5 | 0.000 | | (Ref) |
| *Semaglutide* |  |  |  | |  |
| Pre-exposure period | 0 | 4.4 | 0.000 | | - |
| 0-179 days after initiation | 2 | 4.4 | 0.451 | | 0.572 (0.113-2.900) |
| 180-364 days after initiation | 1 | 4.2 | 0.237 | | 0.309 (0.038-2.513) |
| 365-729 days after initiation | 2 | 4.8 | 0.415 | | 0.645 (0.115-3.619) |
| Non-exposure period | 4 | 4.6 | 0.877 | | (Ref) |
| **Sensitivity analyses** | |  |  | |  |
| *Censor on GLP-1RA discontinuation* | |  |  | |  |
| Pre-exposure period | 10 | 31.0 | 0.322 | | 0.652 (0.295-1.442) |
| 0-179 days after initiation | 14 | 29.3 | 0.478 | | 1.049 (0.502-2.192) |
| 180-364 days after initiation | 5 | 24.6 | 0.204 | | 0.540 (0.189-1.542) |
| 365-729 days after initiation | 18 | 36.6 | 0.492 | | 1.480 (0.679-3.227) |
| Non-exposure period | 16 | 31.6 | 0.507 | | (Ref) |
| *Exclude death cases* |  |  |  | |  |
| Pre-exposure period | 9 | 38.4 | 0.234 | | 0.589 (0.259-1.340) |
| 0-179 days after initiation | 15 | 38.4 | 0.390 | | 1.010 (0.489-2.088) |
| 180-364 days after initiation | 11 | 38.2 | 0.288 | | 0.779 (0.352-1.724) |
| 365-729 days after initiation | 27 | 67.1 | 0.402 | | 1.159 (0.599-2.240) |
| Non-exposure period | 16 | 39.2 | 0.408 | | (Ref) |
| *Exclude depression events on the same day of GLP-1RA initiation* | | | | | |
| Pre-exposure period | 10 | 38.9 | 0.257 | | 0.654 (0.295-1.449) |
| 0-179 days after initiation | 14 | 38.9 | 0.360 | | 0.942 (0.450-1.969) |
| 180-364 days after initiation | 12 | 38.7 | 0.310 | | 0.848 (0.391-1.842) |
| 365-729 days after initiation | 27 | 67.0 | 0.403 | | 1.182 (0.611-2.287) |
| Non-exposure period | 16 | 39.7 | 0.403 | | (Ref) |
| *No pre-exposure period* | | | |  |  |
| 0-179 days after initiation | 15 | 39.4 | 0.381 | | 1.225 (0.640-2.345) |
| 180-364 days after initiation | 12 | 39.2 | 0.306 | | 1.029 (0.509-2.081) |
| 365-729 days after initiation | 27 | 68.0 | 0.397 | | 1.425 (0.802-2.534) |
| Non-exposure period | 26 | 79.6 | 0.327 | | (Ref) |
| *Pre-exposure period of 90 days* |  |  |  | |  |
| Pre-exposure period | 2 | 19.7 | 0.101 | | 0.258 (0.061-1.096) |
| 0-179 days after initiation | 15 | 39.4 | 0.381 | | 0.992 (0.512-1.922) |
| 180-364 days after initiation | 12 | 39.2 | 0.306 | | 0.832 (0.407-1.701) |
| 365-729 days after initiation | 27 | 68.0 | 0.397 | | 1.156 (0.642-2.080) |
| Non-exposure period | 24 | 59.9 | 0.401 | | (Ref) |
| *Adjust for season* |  |  |  | |  |
| Pre-exposure period | 10 | 39.4 | 0.254 | | 0.664 (0.300-1.473) |
| 0-179 days after initiation | 15 | 39.4 | 0.381 | | 1.012 (0.491-2.086) |
| 180-364 days after initiation | 12 | 39.2 | 0.306 | | 0.858 (0.394-1.866) |
| 365-729 days after initiation | 27 | 68.0 | 0.397 | | 1.176 (0.609-2.271) |
| Non-exposure period | 16 | 40.2 | 0.398 | | (Ref) |

**United Kingdom**

|  | **No. of events** | **Follow-up (person-years)** | **Crude incidence (per person-year)** | | **Incident rate ratio (95% CI)** |
| --- | --- | --- | --- | --- | --- |
| **Sex** |  |  |  | |  |
| *Male* |  |  |  | |  |
| Pre-exposure period | 78 | 207.1 | 0.377 | | 0.901 (0.659-1.231) |
| 0-179 days after initiation | 76 | 209.4 | 0.363 | | 0.860 (0.625-1.182) |
| 180-364 days after initiation | 78 | 206.4 | 0.378 | | 0.938 (0.681-1.291) |
| 365-729 days after initiation | 116 | 373.8 | 0.310 | | 0.789 (0.581-1.070) |
| Non-exposure period | 82 | 200.5 | 0.409 | | (Ref) |
| *Female* |  |  |  | |  |
| Pre-exposure period | 69 | 178.2 | 0.387 | | 0.880 (0.632-1.225) |
| 0-179 days after initiation | 68 | 178.2 | 0.382 | | 0.872 (0.624- 1.220) |
| 180-364 days after initiation | 49 | 175.6 | 0.279 | | 0.652 (0.449- 0.947) |
| 365-729 days after initiation | 110 | 317.3 | 0.347 | | 0.824 (0.597- 1.138) |
| Non-exposure period | 73 | 170.5 | 0.428 | | (Ref) |
| **Drug** |  |  |  | |  |
| *Dulaglutide* |  |  |  | |  |
| Pre-exposure period | 20 | 36.2 | 0.552 | | 1.196 (0.623- 2.297) |
| 0-179 days after initiation | 11 | 36.0 | 0.306 | | 0.670 (0.311- 1.444) |
| 180-364 days after initiation | 12 | 34.5 | 0.347 | | 0.800 (0.373- 1.714) |
| 365-729 days after initiation | 14 | 55.3 | 0.253 | | 0.610 (0.288- 1.294) |
| Non-exposure period | 17 | 36.8 | 0.462 | | (Ref) |
| *Exenatide* |  |  |  | |  |
| Pre-exposure period | 53 | 159.2 | 0.333 | | 0.812 (0.557- 1.184) |
| 0-179 days after initiation | 64 | 162.7 | 0.393 | | 0.950 (0.660- 1.366) |
| 180-364 days after initiation | 57 | 163.9 | 0.348 | | 0.851 (0.582- 1.242) |
| 365-729 days after initiation | 102 | 311.4 | 0.328 | | 0.788 (0.556- 1.118) |
| Non-exposure period | 57 | 145.5 | 0.392 | | (Ref) |
| *Liraglutide* |  |  |  | |  |
| Pre-exposure period | 66 | 158.6 | 0.416 | | 1.186 (0.828-1.699) |
| 0-179 days after initiation | 56 | 158.7 | 0.353 | | 1.012 (0.694-1.475) |
| 180-364 days after initiation | 48 | 158.8 | 0.302 | | 0.898 (0.605-1.333) |
| 365-729 days after initiation | 100 | 287.6 | 0.348 | | 1.075 (0.755-1.530) |
| Non-exposure period | 55 | 157.7 | 0.349 | | (Ref) |
| *Lixisenatide* |  |  |  | |  |
| Pre-exposure period | 3 | 13.5 | 0.223 | | 0.282 (0.077-1.030) |
| 0-179 days after initiation | 2 | 13.2 | 0.152 | | 0.189 (0.041-0.882) |
| 180-364 days after initiation | 7 | 12.6 | 0.555 | | 0.725 (0.260-2.026) |
| 365-729 days after initiation | 6 | 22.9 | 0.262 | | 0.368 (0.121-1.123) |
| Non-exposure period | 10 | 13.2 | 0.755 | | (Ref) |
| *Semaglutide* |  |  |  | |  |
| Pre-exposure period | 5 | 17.8 | 0.281 | | 0.285 (0.104-0.785) |
| 0-179 days after initiation | 11 | 17.0 | 0.647 | | 0.613 (0.268-1.399) |
| 180-364 days after initiation | 3 | 12.2 | 0.246 | | 0.266 (0.071-0.989) |
| 365-729 days after initiation | 4 | 14.0 | 0.287 | | 0.323 (0.090-1.158) |
| Non-exposure period | 16 | 17.7 | 0.903 | | (Ref) |
| **Sensitivity analyses** | |  |  | |  |
| *Censor on GLP-1RA discontinuation* | |  |  | |  |
| Pre-exposure period | 147 | 298.2 | 0.493 | | 0.893 (0.711- 1.121) |
| 0-179 days after initiation | 130 | 280.2 | 0.464 | | 0.869 (0.683- 1.104) |
| 180-364 days after initiation | 85 | 227.9 | 0.373 | | 0.794 (0.599- 1.052) |
| 365-729 days after initiation | 101 | 318.7 | 0.317 | | 0.716 (0.536- 0.957) |
| Non-exposure period | 155 | 288.2 | 0.538 | | (Ref) |
| *Exclude death cases* |  |  |  | |  |
| Pre-exposure period | 146 | 382.8 | 0.381 | | 0.889 (0.708-1.117) |
| 0-179 days after initiation | 144 | 385.1 | 0.374 | | 0.870 (0.690-1.096) |
| 180-364 days after initiation | 124 | 379.7 | 0.327 | | 0.787 (0.617-1.004) |
| 365-729 days after initiation | 226 | 689.3 | 0.328 | | 0.805 (0.644-1.005) |
| Non-exposure period | 154 | 368.4 | 0.418 | | (Ref) |
| *Exclude depression events on the same day of GLP-1RA initiation* | | | | | |
| Pre-exposure period | 147 | 380.2 | 0.387 | | 0.891 (0.710-1.118) |
| 0-179 days after initiation | 133 | 382.5 | 0.348 | | 0.800 (0.632-1.013) |
| 180-364 days after initiation | 127 | 377.0 | 0.337 | | 0.801 (0.629-1.019) |
| 365-729 days after initiation | 226 | 683.1 | 0.331 | | 0.799 (0.640-0.998) |
| Non-exposure period | 155 | 365.9 | 0.424 | | (Ref) |
| *No pre-exposure period* | | | |  |  |
| 0-179 days after initiation | 144 | 387.6 | 0.372 | | 0.919 (0.751-1.123) |
| 180-364 days after initiation | 127 | 382.1 | 0.332 | | 0.852 (0.689-1.055) |
| 365-729 days after initiation | 226 | 691.2 | 0.327 | | 0.855 (0.708-1.034) |
| Non-exposure period | 302 | 756.2 | 0.399 | | (Ref) |
| *Pre-exposure period of 90 days* |  |  |  | |  |
| Pre-exposure period | 72 | 195.2 | 0.369 | | 0.873 (0.669-1.139) |
| 0-179 days after initiation | 144 | 387.6 | 0.372 | | 0.887 (0.717-1.096) |
| 180-364 days after initiation | 127 | 382.1 | 0.332 | | 0.823 (0.658-1.028) |
| 365-729 days after initiation | 226 | 691.2 | 0.327 | | 0.825 (0.675-1.009) |
| Non-exposure period | 230 | 561.0 | 0.410 | | (Ref) |
| *Adjust for season* |  |  |  | |  |
| Pre-exposure period | 147 | 385.2 | 0.382 | | 0.893 (0.712-1.121) |
| 0-179 days after initiation | 144 | 387.6 | 0.372 | | 0.866 (0.687-1.091) |
| 180-364 days after initiation | 127 | 382.1 | 0.332 | | 0.806 (0.633-1.027) |
| 365-729 days after initiation | 226 | 691.2 | 0.327 | | 0.810 (0.649-1.010) |
| Non-exposure period | 155 | 371.0 | 0.418 | | (Ref) |

**Taiwan**

|  | | **No. of events** | **Follow-up (person-years)** | | **Crude incidence (per person-year)** | | | **Incident rate ratio (95% CI)** |  |
| --- | --- | --- | --- | --- | --- | --- | --- | --- | --- |
| **Sex** | |  |  | |  | | |  |  |
| *Male* | |  |  | |  | | |  |  |
| Pre-exposure period | | 94 | 244.4 | | 0.385 | | | 1.058 (0.775- 1.444) |  |
| 0-179 days after initiation | | 101 | 234.8 | | 0.430 | | | 1.354 (0.930- 1.972) |  |
| 180-364 days after initiation | | 85 | 231.4 | | 0.367 | | | 1.287 (0.801- 2.066) |  |
| 365-729 days after initiation | | 133 | 392.1 | | 0.339 | | | 1.380 (0.751- 2.534) |  |
| Non-exposure period | | 94 | 239.2 | | 0.393 | | | (Ref) |  |
| *Female* | |  |  | |  | | |  |  |
| Pre-exposure period | | 163 | 399.6 | | 0.408 | | | 0.967 (0.763- 1.224) |  |
| 0-179 days after initiation | | 149 | 389.3 | | 0.383 | | | 0.975 (0.727- 1.309) |  |
| 180-364 days after initiation | | 126 | 384.8 | | 0.328 | | | 0.897 (0.618- 1.302) |  |
| 365-729 days after initiation | | 218 | 666.4 | | 0.327 | | | 0.997 (0.617- 1.609) |  |
| Non-exposure period | | 170 | 385.9 | | 0.441 | | | (Ref) |  |
| **Drug** | |  |  | |  | | |  |  |
| *Dulaglutide* |  | | |  | |  | |  | |
| Pre-exposure period | 113 | | | 282.2 | | 0.401 | | 0.939 (0.708- 1.246) | |
| 0-179 days after initiation | 110 | | | 266.4 | | 0.413 | | 1.053 (0.743- 1.493) | |
| 180-364 days after initiation | 96 | | | 254.1 | | 0.378 | | 1.046 (0.670- 1.633) | |
| 365-729 days after initiation | 131 | | | 399.2 | | 0.328 | | 1.000 (0.563- 1.776) | |
| Non-exposure period | 127 | | | 282.2 | | 0.450 | | (Ref) | |
| *Liraglutide* |  | | |  | |  | |  | |
| Pre-exposure period | 142 | | | 358.8 | | 0.396 | | 1.068 (0.827- 1.380) | |
| 0-179 days after initiation | 139 | | | 356.9 | | 0.390 | | 1.152 (0.842- 1.576) | |
| 180-364 days after initiation | 115 | | | 362.0 | | 0.318 | | 1.014 (0.685- 1.502) | |
| 365-729 days after initiation | 220 | | | 659.3 | | 0.334 | | 1.222 (0.740- 2.019) | |
| Non-exposure period | 134 | | | 339.9 | | 0.394 | | (Ref) | |
| *Semaglutide* |  | | |  | |  | |  | |
| Pre-exposure period | 2 | | | 2.9 | | 0.680 | | 0.778 (0.108- 5.581) | |
| 0-179 days after initiation | 1 | | | 0.8 | | 1.209 | | 1.571 (0.123- 20.104) | |
| 180-364 days after initiation | 0 | | | 0.0 | | 0.000 | | - | |
| 365-729 days after initiation | 0 | | | 0.0 | | 0.000 | | - | |
| Non-exposure period | 3 | | | 2.9 | | 102.430 | | (Ref) | |
| **Sensitivity analyses*** | | |  | |  | | |  |  |
| *Censor on GLP-1RA discontinuation* | | |  | |  | | |  |  |
| Pre-exposure period | | 257 | 643.9 | | 0.389 | | | 1.002 (0.831- 1.210) |  |
| 0-179 days after initiation | | 250 | 637.8 | | 0.382 | | | 1.064 (0.845- 1.340) |  |
| 180-364 days after initiation | | 211 | 626.7 | | 0.328 | | | 0.998 (0.745- 1.338) |  |
| 365-729 days after initiation | | 351 | 1058.5 | | 0.323 | | | 1.121 (0.769- 1.632) |  |
| Non-exposure period | | 264 | 625.0 | | 0.412 | | | (Ref) |  |
| *Exclude death cases* | |  |  | |  | | |  |  |
| Pre-exposure period | | 235 | 601.7 | | 0.391 | | | 1.003 (0.824- 1.220) |  |
| 0-179 days after initiation | | 237 | 596.9 | | 0.397 | | | 1.103 (0.869- 1.401) |  |
| 180-364 days after initiation | | 199 | 589.4 | | 0.338 | | | 1.021 (0.755- 1.382) |  |
| 365-729 days after initiation | | 332 | 997.6 | | 0.333 | | | 1.147 (0.778- 1.691) |  |
| Non-exposure period | | 242 | 584.8 | | 0.414 | | | (Ref) |  |
| *No pre-exposure period* | | | | | | |  |  |  |
| 0-179 days after initiation | | 250 | 624.1 | | 0.401 | | | 1.104 (0.918- 1.328) |  |
| 180-364 days after initiation | | 211 | 616.1 | | 0.343 | | | 1.026 (0.808- 1.303) |  |
| 365-729 days after initiation | | 351 | 1058.5 | | 0.332 | | | 1.124 (0.825- 1.531) |  |
| Non-exposure period | | 521 | 1269.0 | | 0.411 | | | (Ref) |  |
| *Pre-exposure period of 90 days* | |  |  | |  | | |  |  |
| Pre-exposure period | | 127 | 322.7 | | 0.394 | | | 0.998 (0.807- 1.236) |  |
| 0-179 days after initiation | | 250 | 637.8 | | 0.392 | | | 1.060 (0.863- 1.302) |  |
| 180-364 days after initiation | | 211 | 626.7 | | 0.337 | | | 0.993 (0.761- 1.295) |  |
| 365-729 days after initiation | | 351 | 1058.5 | | 0.332 | | | 1.112 (0.787- 1.571) |  |
| Non-exposure period | | 394 | 946.2 | | 0.416 | | | (Ref) |  |
| *Adjust for season* | |  |  | |  | | |  |  |
| Pre-exposure period | | 257 | 643.9 | | 0.399 | | | 0.957 (0.786- 1.165) |  |
| 0-179 days after initiation | | 250 | 624.1 | | 0.401 | | | 0.976 (0.755- 1.261) |  |
| 180-364 days after initiation | | 211 | 616.1 | | 0.343 | | | 0.916 (0.654- 1.284) |  |
| 365-729 days after initiation | | 351 | 1058.5 | | 0.332 | | | 0.958 (0.609- 1.506) |  |
| Non-exposure period | | 264 | 625.0 | | 0.422 | | | (Ref) |  |

* No depression event occurred on the same day as GLP1-RA initiation.

**Supplementary Figure 1. Flowchart for selection of SCCS cases**

**Hong Kong**

Incident depression cases aged ≥18 years with GLP-1RA initiation during the study period
 (n=549)

Cases included in analyses (n=80)

Exclude depression events outside observation period:

- Depression occurred >1 year before or >2 years after GLP1-RA initiation (n=469)

**United Kingdom**

Incident depression cases aged ≥18 years with GLP-1RA initiation during the study period

(n=3,364)

Cases included in analyses (n=799)

Exclude depression events outside observation period:

- Depression occurred >1 year before or >2 years after GLP1-RA initiation (n=2,565)

**Taiwan**

Incident depression cases aged ≥18 years with GLP-1RA initiation during the study period (n=3,618 [exclude the cases with missing sex])

Cases included in analyses (n=1,333)

Exclude depression events outside observation period:

- Depression occurred >1 year before or >2 years after GLP1-RA initiation (n=2,285)

**Supplementary Figure 2. Study design**

Non-exposure period

Pre-exposure

Day 0-179

Day 180-364

Day 365-729

Observation period

Start date of first-ever GLP-1RA prescription

Latest of:

- 1 year before GLP-1RA initiation
- start of study period

Earliest of:

- 2 years after GLP-1RA initiation
- date of death
- end of study period

Incident diagnosis of depression
